# 

**Published:** 1915-01-23

**Authors:** 


### CONTENTS.

PAGE

**The War and Medical Teaching** ... ... ... 365

**Hospital Policy During the War** ... ... 366

**Hospital and Institutional News** ... ... ... 367

The Chadwick Prize for Navy and Army Doctors—Three Months' Contracts for Poor-Law Infirmaries — Rights of Civilians at Halifax Royal Infirmary—Soldiers in Hospital: An Official View—The Alternative to Hospitals for the Wounded—A Hospital Ship for Servia—A Patriotic Institution—London's Mental Hospital Work—Tribute to a Red Cross Secretary—The West Derby Guardians and Medical Salaries—The Fire at Dursley Infectious Hospital—A Tuberculosis Dispensary for Men in the City—The Cases at Claybury—The Profession and Medical Trade Unionism—The "Luxury" of Institutional Officers—A Minister and Thrift on 8s. a Week—A Red Cross Grievance in Scotland—Torquay Hospitals and the Wounded—An Improvement in the Annual Report—Public Gardens as a Military Hospital—The Manufacture of Synthetic Drugs and Aniline Dyes—The New Manchester Union—This Week's Drug Market.

**Surgical Experiences in the Present War** ... 373

Anti-Typhoid Inoculation in the Field.

**War and Disease** ... ... ... ... ... 375

Continental Wars of the Nineteenth Century.

**Hospitals and Smoke** ... ... ... ... 376

PAGE

**Hospital Work in Egypt** ... ... ... ... 377

The Ophthalmic Problem for Hospitals and Doctors.

**Institutional Organisation in America** ... ... 378

The Neurological Institute in New York City.

**The Clinical Value of Poor-Law Infirmaries** 379

Their Lack of System and Suggested Reforms.

**Round the World** ... ... ... ... ... 380

Fellow-Workers and the Roll of Honour.

**The Cost of Hospitals with Medical Schools**... 381

King Edward's Hospital Fund for London Statistics—Third Notice.

**Economy in the Generation and Use of Steam** 382

Waste of Heat in the Boiler.

**India-Rubber Flooring for Hospital Work** ... 383

**The Editor's Letter-Box** ... ... ... ... 385

The Roll of Honour of the Royal College of Surgeons in Ireland—The War and the Rainfall.

**The Hospital Sunday Fund** ... ... ... 385

Election of Vice-Chairman and Officers.

**Some Provincial Hospital Chapels** ... ... 386

**Medical Appointments** ... ... ... ... 388

**Personalia** ... ... ... ... ... ... 388

**The Institutional Worker** ... ... (Suppl.) 1

Radiography and Some of its Difficulties.
Question for January.
Questions Invited.

